# Comparison of the Efficacy and Safety of a Doravirine-Based, Three-Drug Regimen in Treatment-Naïve HIV-1 Positive Adults: A Bayesian Network Meta-Analysis

**DOI:** 10.3389/fphar.2022.676831

**Published:** 2022-04-20

**Authors:** Ke Zhang, Yang Zhang, Jing Zhou, Lulu Xu, Chi Zhou, Guanzhi Chen, Xiaojie Huang

**Affiliations:** ^1^ Department of Dermatology, The Affiliated Hospital of Qingdao University, Qingdao, China; ^2^ Clinical and Research Center for Infectious Diseases, Beijing Youan Hospital, Capital Medical University, Beijing, China; ^3^ Beijing Key Laboratory of HIV/AIDS Research, Beijing Youan Hospital, Capital Medical University, Beijing, China; ^4^ Infectious Disease Department, University of Chinese Academy of Sciences Shenzhen Hospital (Guangming), Shenzhen, China

**Keywords:** HIV, antiretroviral therapy, randomized controlled trials, doravirine, network meta-analysis

## Abstract

**Introduction:** Extensive use of antiretroviral therapy has remarkably improved the survival rates of people living with HIV. Doravirine (DOR) is a newly-approved antiretroviral belonging to the class of non-nucleoside reverse transcriptase inhibitors. Here, we compared the efficacy and safety of DOR + tenofovir dipivoxil fumarate (TDF)+Lamivudine (3TC)/Emtritabine (FTC) with traditional triple therapies in treatment-naïve HIV-1-positive adults.

**Methods:** Randomized controlled trials involving treatment-naïve HIV-1-positive adults that met inclusion criteria were systematically retrieved and data on the following outcomes extracted: virological suppression, adverse events, severe adverse events, and drug-related adverse events. A Bayesian network meta-analysis was then performed on the data.

**Results:** This study included a total of 39 randomized controlled trials involving 26 antiretroviral therapies and 21,110 HIV1-positive patients. At week 48, relative to the other 25 regimens included in the network of virological suppression, DOR + TDF+3TC/FTC exhibited superiority to some efavirenz, nevirapine, atazanavir, or lopinavir-based regimens, including efavirenz + abacavir+3TC [Odd Ratio (OR) = 0.52, 95% confidence interval (CrI) = 0.35–0.77]. At week 48, the performance of DOR + TDF+3TC/FTC was relatively similar to all other analyzed regimens in terms of adverse events. The DOR + TDF+3TC/FTC regimen performed better in terms of severe adverse events and drug-related adverse events.

**Conclusion:** The network meta-analysis showed that DOR + TDF+3TC/FTC has good efficacy and safety at 48 weeks.

**Systematic Review Registration:** Open Science Framework, https://osf.io/6ybp7.

## Introduction

HIV is the causative agent of AIDS. In 2020, there were 37.7 million (30.2 million–45.1 million) people living with HIV (PLWH) (UNAIDS, 2020). HIV-1, a HIV subtype is the main driver of the global AIDS pandemic. Studies have shown that antiretroviral therapy (ART) has significantly reduced the rates of HIV-1-induced, AIDS-related, morbidity and mortality, and the life expectancy of HIV-1-infected people is nearing that of the general population (Antiretroviral Therapy Cohort Collaboration, 2017; Wu, 2016; Marcus et al., 2000; GBD 2019 HIV Collaborators, 2021; Smiley et al., 2021). Regimens for initiating antiretroviral therapy in HIV-infected people are usually comprised of two nucleoside reverse transcriptase inhibitors as backbone drugs, combined with a third core drug. The core drugs may be non-nucleoside reverse transcriptase inhibitors (NNRTIs) or boosted protease inhibitors or integrase inhibitors (European AIDS Clinical Society, 2019; Panel on Antiretroviral Guidelines for Adults and Adolescents, 2021).

Doravirine (DOR), a new FDA-approved NNRTI is currently prescribed in combination with tenofovir disoproxil fumarate (TDF) and lamivudine (3TC) in the US, China, Africa and et al. (Rock et al., 2020; Panel on Antiretroviral Guidelines for Adults and Adolescents, 2021; Steegen et al., 2021). Two phase 3 clinical trials (DRIVE-AHEAD, DRIVE-FORWARD), have compared DOR + TDF+3TC/emtricitabine (FTC) with efavirenz (EFV)- or darunavir (DRV)-based regimens in treatment-naïve, HIV-1-positive patients (Molina et al., 2018; Orkin et al., 2019) and found that it is efficacious, safe and tolerable. Since the emergence of ART, the survival rate of HIV- infected people has improved significantly, turning HIV infection into a controllable chronic disease (Antiretroviral Therapy Cohort Collaboration, 2017; Wu, 2016; Marcus et al., 2000; GBD 2019 HIV Collaborators, 2021; Smiley et al., 2021). However, PLWH often need to use ART for life, and as their life expectancy increases, so does the amount of time they are on ART. Thus, ART toxicity has attracted considerable attention in clinical practice. In the case of similar efficacy, treatment regimens with less toxicity and high safety are preferred. However, due to the time-consuming and labor-intensive nature of clinical trials, DOR + TDF+3TC/FTC has not been directly compared with all first-line ARTs in randomized controlled trials (RCT). Hence, the efficacy and safety of this regimen relative to other regimens has not been directly determined. We found that network meta-analysis (NMA) is increasingly used to support the development of treatment guidelines. For example, a 2016 NMA evaluated the use of core drugs in untreated HIV-1-positive people, and found that integrase inhibitors, especially DTG, showed excellent effectiveness and safety (Kanters et al., 2016).

Here, in the absence of direct comparisons, we sought to evaluate the effectiveness and safety of various ARTs by indirect comparison. NMA combines direct and indirect evidence to simultaneously assess the relative effectiveness and safety of two or more interventions. Our objective was to compare the efficacy and safety of DOR + TDF+3TC/FTC with traditional three-drug regiments at 48 weeks in treatment-naïve HIV-1-infected adults.

## Methods

### Study Identification and Selection Criteria

The current NMA was conducted on the basis of the PRISMA extension statement (Hutton et al., 2015).

In November 2020 and February 2022, we systematically searched PubMed, embase, Web of Science, and Cochrane Library databases for RCTs meeting inclusion criteria. Search strategy can be found in the protocol at https://osf.io/6ybp7. Inclusion criteria were as follows: 1) phase 3 or 4 RCTs of treatment-naïve HIV-1-infected adults, 2) interventions: DOR + TDF+3TC/FTC or other ART regimens with three antiretrovirals that included 2 NRTIs (backbones) and one core agent. Some core drugs need the assistance of boosted drugs (b) like cobicistat (c) and ritonavir (r) to enhance their effect. The following cores and backbones were chosen because they were used or are still used as first-line therapies. Although some regimens may no longer be used as often, we also included these regimens to serve as comparators in order to increase the connectivity of the network, provide more indirect effects. The backbones of our interest were TDF+3TC/FTC, tenofovir alafenamide (TAF)+FTC, abacavir (ABC)+3TC, or azidothymidine (AZT)+3TC. Core drugs of our interest were raltegravir (RAL), elvitegravir (EVG), dolutegravir (DTG), bictegravir (BIC), EFV, rilpivirine (RPV), nevirapine (NVP), atazanavir (ATV), lopinavir (LPV), and DRV, 3) all included drugs were standard doses, but low-dose EFV (400mg EFV) were also included, 4) at least one of the following outcomes provided: 48-weeks virological suppression (HIV-1 RNA<50 copies/mL), adverse events, serious adverse events, drug-related adverse events, subgroup analysis (virological suppression in people with a baseline viral load of >100,000 copies/mL). Exclusion criteria were: 1) all subjects living with tuberculosis or were pregnant, 2) inability to precisely determine the backbones used. To avoid missing relevant data, we searched www.ClinicalTrials.gov and www.napnap.com, and we also read relevant systematic reviews, meta-analyses, and their references.

Past studies used third core drugs as network nodes for analysis when comparing the effectiveness and safety of ARTs in treatment-naive HIV patients (Kanters et al., 2016; Gallien et al., 2018). Considering that the backbone of the DOR + TDF+3TC/FTC we wanted to compare is defined, we used a complete treatment regimen as network node. Analyzing a comprehensive treatment regimen as a network node can also simultaneously evaluate differences between regimens of the same core drug with different backbones, thereby providing more reference for clinical treatment.

### Data Extraction and Quality Evaluation

Two researchers (KZ and YZ) independently selected titles and abstracts, read full texts of articles that met the inclusion criteria, and extracted relevant data (Supplementary Table S1). Any disagreements were resolved by discussion and if no solution was reached, the opinion of the third researcher (JZ) was taken. The Cochrane Risk of Bias instrument was used to assess quality of included trials (Supplementary Table S2) (Higgins et al., 2011).

### Analysis

R software (version 4.0.2) and gemtc package were used to perform NMA within a Bayesian framework. Results were calculated using Markov chain Monte Carlo method, and the convergence evaluated using a potential scale reduction factor (<1.2 is acceptable) (Shim et al., 2019). The results of fixed effect model and random effect model were calculated in this paper. By comparing deviance information criterion of the fixed effect and random effect models, the more suitable model was selected. The smaller the deviance information criterion, the more appropriate the model was. The consistency test was analysed by node splitting method, with *p* < 0.05 indicating inconsistency. The random-effect model was used when local comparisons were inconsistent. In case of inconsistency in many comparisons, the suitability of NMA should be discussed (Shim et al., 2019). The outcomes analyzed in this study were all binary variables, and the statistical results were expressed as odds ratio (OR) and corresponding 95% confidence interval (CrI). SUCRA values for each regimen were calculated to evaluate their ranking among various regimens. CINeMA was used to grade evidence quality (Nikolakopoulou et al., 2020).

## Results

### Studies Included

**FIGURE 1:**
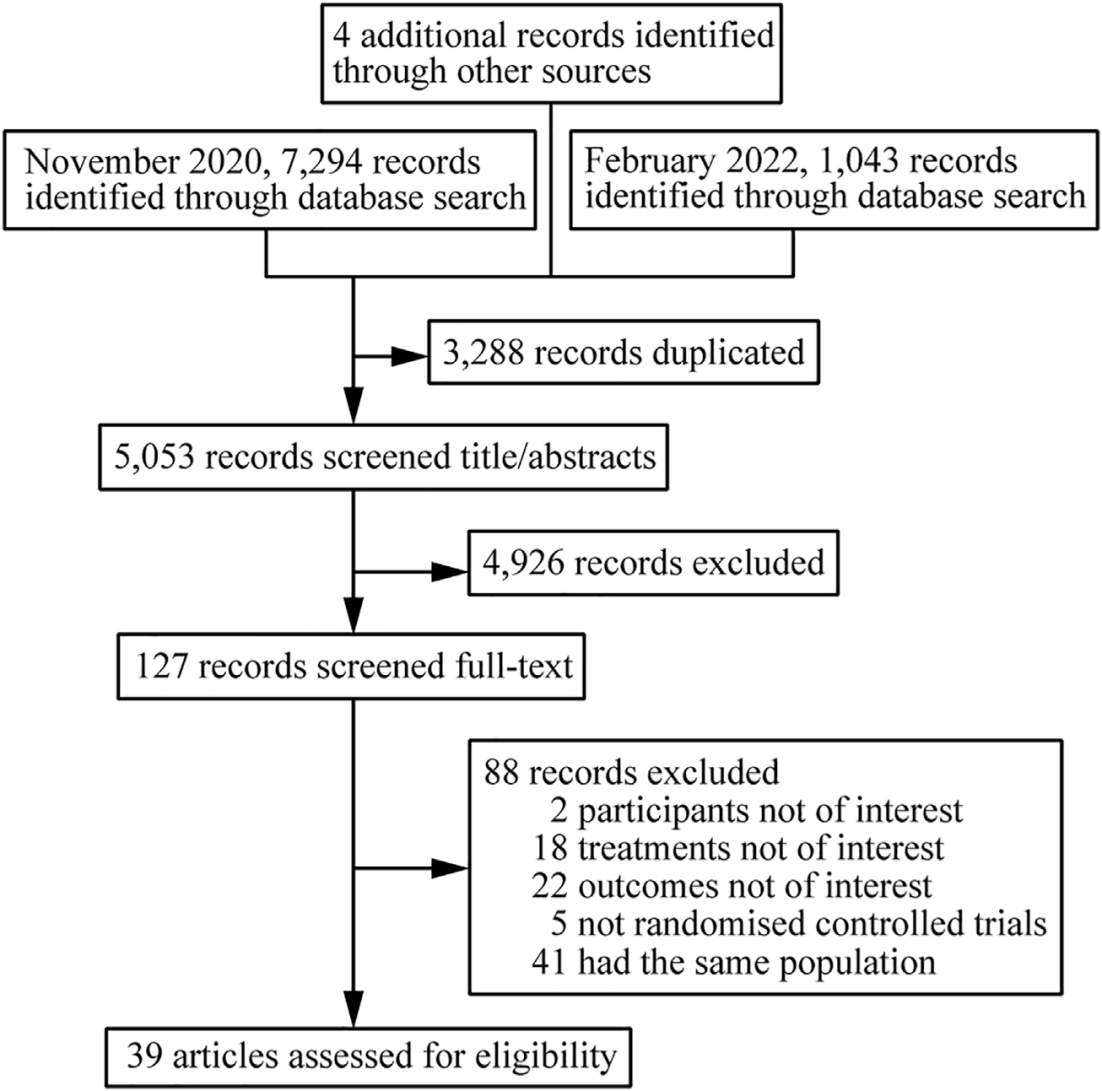
Flow chart of study selection.

**FIGURE 2:**
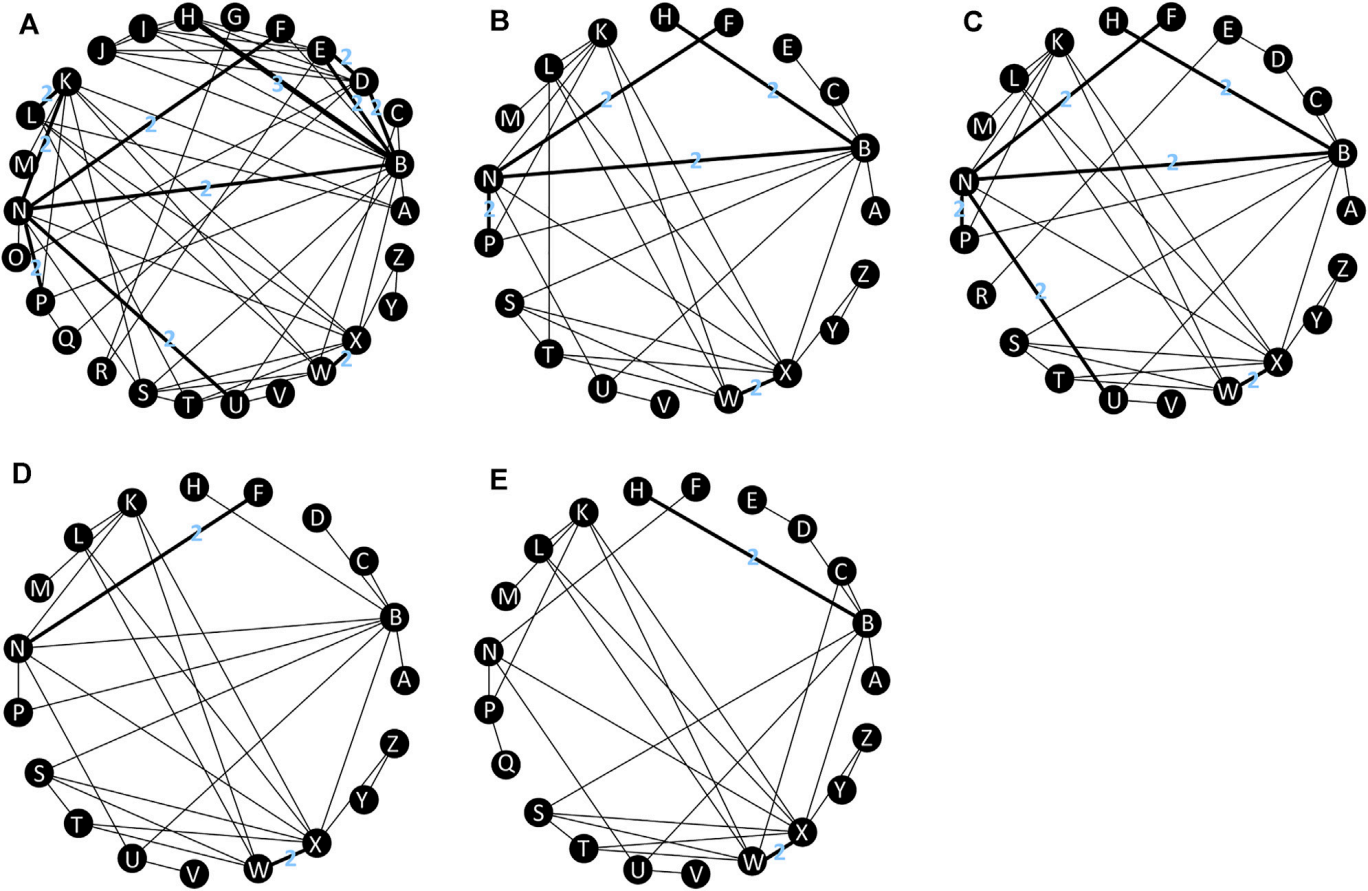
Network plot of treatment outcomes in terms of **(A)** virological suppression, **(B)** adverse events, **(C)** serious adverse events, **(D)** drug-related adverse events, **(E)** subgroup analysis. All connecting straight lines are based either on single studies or in case this does not hold true, then every connection with *n* > 1 should show the actual n. A: DOR + TDF + XTC; B: EFV + TDF + XTC; C: EFV400 + TDF + XTC; D: EFV + ABC+3TC; E: EFV + AZT+3TC; F: NVP + TDF + XTC; G: NVP + AZT+3TC; H: RPV + TDF + XTC; I: RPV + ABC+3TC; J: RPV + AZT+3TC; K: DRV/b + TDF + XTC; L: DRV/r + ABC+3TC; M: DRV/c + TAF + FTC; N: ATV/r + TDF + XTC; O: ATV/r + ABC+3TC; P: LPV/r + TDF + XTC; Q: LPV/r + ABC+3TC; R: LPV/r + AZT+3TC; S: RAL + TDF + XTC; T: RAL + ABC+3TC; U: EVG/c + TDF + XTC; V: EVG/c + TAF + FTC; W: DTG + TDF + XTC; X: DTG + ABC+3TC; Y: DTG + TAF + FTC; Z: BIC + TAF + FTC. ABC: abacavir; ATV: atazanavir; AZT: Azidothymidine; BIC: bictegravir; c: cobicistat; DOR: doravirine; DRV: darunavir; DTG: dolutegravir; EFV: efavirenz; EVG: elvitegravir; FTC: emtricitabine; LPV: lopinavir; NVP: nevirapine; r: ritonavir; RAL: raltegravir; RPV: rilpivirine; TAF: tenofovir alafenamide; TDF: tenofovir disoproxil fumarate; XTC: emtricitabine or lamivudine; 3TC: lamivudine.

A total of 8341 citations were obtained using database searches and other methods. Of these, 3288 duplicates were excluded. After screening, 39 articles (Orkin et al., 2019; Molina et al., 2018; Lennox et al., 2014; Miro et al., 2015; Puls et al., 2010; Eron et al., 2018; Orrell et al., 2017; Ortiz et al., 2008; Soriano et al., 2011; Post et al., 2010; Molina et al., 2008; DeJesus et al., 2004; Harris et al., 2009; Landman et al., 2014; Molina et al., 2011; ENCORE1 Study Group, 2014; Nishijima et al., 2013; Clotet et al., 2014; Sax et al., 2012; DeJesus et al., 2012; Sax et al., 2015; Gallant et al., 2017; Sax et al., 2017; Smith et al., 2009; Honda et al., 2011; Echeverría et al., 2010; Aberg et al., 2012; Kouanfack et al., 2019; Dejesus et al., 2011; Mussini et al., 2019; Sierra-Madero et al., 2010; Walmsley et al., 2013; Raffi et al., 2013; Cohen et al., 2014; Lennox et al., 2009; Gallant et al., 2006; Cohen et al., 2012; Cohen et al., 2011; Squires et al., 2016), involving 39 RCTs and 21,110 participants, met the inclusion criteria (Figure 1). The number of participants in different studies ranged from 46 to 1,809, and most of the studies involved predominantly male participants. In different studies, baseline characteristics like the mean/median age, CD4 and viral load of participants in different treatment groups were 32.7–41 years, 30 to 476 cells/mm^3^, 4.28 to 5.6 log copies/mL respectively. Table 1 shows the basic information for each trial and the baseline characteristics of the study population. The ARTs analyzed for each outcome are plotted in network plots, with straight line connections between the different ARTs indicating the existence of a RCT that directly compared the 2 ARTs (Figure 2). Fixed-effect model was preferred for all the analysis, except analysis in adverse events.

**TABLE 1:** Information of the trials and baseline characteristics of participants.

Study ID	Treatments	Population	Age (years)Mean	Male %	CD4 (Cells/mm3) mean	VL (Log Copies/mL) mean
ACTG A5257 (Lennox et al., 2014)	ATV/r + TDF + FTC	605	38	76.2%	309	4.6
	RAL + TDF + FTC	603	37	75.5%	306	4.6
	DRV/r + TDF + FTC	601	38	76.2%	310	4.6
Advanz-3 (Miro et al., 2015)	EFV + TDF + FTC	28	39^a^	72.4%	41^a^	5.12^a^
	ATV/r + TDF + FTC	30	38.5^a^	90%	32^a^	5.48^a^
	LPV/r + TDF + FTC	29	36.5^a^	83.3%	30^a^	5.15^a^
ALTAIR (Puls et al., 2010)	EFV + FTC + TDF	114	37.3	79.1%	227	4.67
	ATV/r + TDF + FTC	105	36.7	72.0%	235	4.77
AMBER (Eron et al., 2018)	DRV/c + TAF + FTC	362	35.6	87.8%	461.5^a^	4.44^a^
	DRV/c + TDF + FTC	363	35.1	88.7%	440.0^a^	4.57^a^
ARIA (Orrell et al., 2017)	DTG + ABC+3TC	248	38.1	0%	340	4.41
	ATV/r + TDF + FTC	247	37.8	0%	350	4.43
ARTEMIS (Ortiz et al., 2008)	DRV/r + TDF + FTC	343	35.5	69.7%	228^a^	4.86
	LPV/r + TDF + FTC	346	35.3	69.7%	218^a^	4.84
ARTEN (Soriano et al., 2011)	NVP + TDF + FTC	376	39	83.8%	182	5.1
	ATV/r + TDF + FTC	193	38	83.9%	188	5.1
ASSERT (Post et al., 2010)	EFV + ABC+3TC	192	38^a^	82.8%	240^a^	5.01^a^
	EFV + TDF + FTC	193	36^a^	79.3%	230^a^	5.12^a^
CASTLE (Molina et al., 2008)	ATV/r + TDF + FTC	440	36	68.6%	205^a^	5.01^a^
	LPV/r + TDF + FTC	443	37	68.6%	204^a^	4.96^a^
CNA30024 (DeJesus et al., 2004)	EFV + AZT+3TC	325	35^a^	82.2%	258^a^	4.76^a^
	EFV + ABC+3TC	324	35^a^	79.6%	267^a^	4.81^a^
CTN177 (Harris et al., 2009)	LPV/r + AZT+3TC	25	37^a^	75%	210^a^	--
	NVP + AZT+3TC	26				
DAYANA (Landman et al., 2014)	NVP + TDF + FTC	31	37^a^	45.2%	191^a^	5.4^a^
	EFV + TDF + FTC	30	40^a^	26.7%	201^a^	5.6^a^
DRIVE AHEAD (Orkin et al., 2019)	DOR + TDF+3TC	364	33.6	83.8%	434.9	4.4^a^
	EFV + TDF + FTC	364	32.7	85.4%	415.5	4.5^a^
DRIVE FORWARD (Molina et al., 2018)	DOR+(TDF + FTC) or (ABC+3TC)^b^	383	34.8	83.3%	432.6	4.4
	DRV/r+(TDF + FTC) or (ABC+3TC)	383	35.7	85.1%	411.9	4.4
ECHO (Molina et al., 2011)	RPV + TDF + FTC	346	37	77.5%	240^a^	5.0^a^
	EFV + TDF + FTC	344	36.7	79.9%	257^a^	5.0^a^
ENCORE1 (ENCORE1 Study Group, 2014)	EFV400 + TDF + FTC	321	36.1	68.8%	273	4.76^a^
	EFV + TDF + FTC	309	35.8	66.7%	272	4.73^a^
Epzicom-Truvada (Nishijima et al., 2013)	ATV/r + TDF + FTC	55	35^a^	98.2%	269^a^	4.28^a^
	ATV/r + ABC+3TC	54	39^a^	98.1%	236.5^a^	4.29^a^
FLAMINGO (Clotet et al., 2014)	DTG+(TDF + FTC) or (ABC+3TC)	242	35.7	87.2%	390^a^	4.49^a^
	DRV/r+(TDF + FTC) or (ABC+3TC)	242	36.2	83.1%	400^a^	4.48^a^
GS-US-236-0102 (Sax et al., 2012)	EVG/c + TDF + FTC	348	38	88.2%	391	4.73
	EFV + TDF + FTC	352	38	89.8%	382	4.78
GS-US-236-0103 (DeJesus et al., 2012)	EVG/c + TDF + FTC	353	38	91.8%	351^a^	4.8
	ATV/r + TDF + FTC	355	39	89.0%	366^a^	4.8
GS-US-292-0104/0111 (Sax et al., 2015)	EVG/c + TAF + FTC	866	33^a^	84.6%	404^a^	4.58^a^
	EVG/c + TDF + FTC	867	35^a^	85.4%	406^a^	4.58^a^
GS-US-380-1489 (Gallant et al., 2017)	DTG + ABC+3TC	315	34	89.5%	476	4.42
	BIC + TAF + FTC	314	34	90.8%	453	4.41
GS-US-380-1490 (Sax et al., 2017)	BIC + TAF + FTC	320	37	87.5%	457	4.39
	DTG + TAF + FTC	325	37	88.6%	454	4.42
HEAT (Smith et al., 2009)	LPV/r + ABC+3TC	343	38.0	83.7%	214^a^	4.90^a^
	LPV/r + TDF + FTC	345	38.7	80.0%	193^a^	4.84^a^
Japanese Anti-HIV-1 QD Therapy (Honda et al., 2011)	EFV + ABC+3TC	36	35^a^	100%	220^a^	4.6^a^
	ATV/r + ABC+3TC	35	36^a^	100%	226^a^	4.4^a^
LAKE Study (Echeverría et al., 2010)	EFV + ABC+3TC	63	39	86.0%	193	5.4
	LPV/r + ABC+3TC	63	37	86.8%	191	5.3
METABOLIK (Aberg et al., 2012)	DRV/r + TDF + FTC	34	36.5^a^	85.3%	267^a^	5.0^a^
	ATV/r + TDF + FTC	31	35^a^	87.1%	316^a^	4.6^a^
NAMSAL (Kouanfack et al., 2019)	DTG + TDF+3TC	310	38^a^	36.5%	289^a^	5.3^a^
	EFV400 + TDF+3TC	303	36^a^	31.7%	271^a^	5.3^a^
NEWART (Dejesus et al., 2011)	NVP + TDF + FTC	75	37.7	86.7%	178.9	4.9
	ATV/r + TDF + FTC	77	35.9	92.2%	183.5	4.9
PRADAR (Mussini et al., 2019)	DRV/r + ABC+3TC	24	35^a^	79.2%	107^a^	--
	RAL + ABC+3TC	22	41^a^	86.4%	108^a^	--
Sierra-Madero et al., 2010 (Sierra-Madero et al., 2010)	EFV + AZT+3TC	95	36.7^a^	83.2%	64^a^	--
	LPV/r + AZT+3TC	94	36^a^	87.2%	52^a^	--
SINGLE (Walmsley et al., 2013)	DTG + ABC+3TC	414	36.5	83.8%	334.5^a^	4.67^a^
	EFV + TDF + FTC	419	36.4	85.0%	339^a^	4.7^a^
SPRING-2 (Raffi et al., 2013)	DTG+(TDF + FTC) or (ABC+3TC)	411	37.3	84.7%	359^a^	4.52^a^
	RAL+(TDF + FTC) or (ABC+3TC)	411	36.6	86.4%	362^a^	4.58^a^
STaR (Cohen et al., 2014)	RPV + TDF + FTC	394	37	92.9%	395.7	4.8
	EFV + TDF + FTC	392	37	92.9%	385.2	4.8
STARTMRK (Lennox et al., 2009)	RAL + TDF + FTC	281	37.6	80.9%	218.9	5
	EFV + TDF + FTC	282	36.9	82.2%	217.4	5
Study 934 (Gallant et al., 2006)	EFV + TDF + FTC	255	38	85.9%	246	5.03
	EFV + AZT+3TC	254	38	87.0%	245	5.00
THRIVE (Cohen et al., 2012; Cohen et al., 2011)	RPV+(TDF + FTC) or (ABC+3TC) or (AZT+3TC)	340	36.7	73.5%	263^a^	5.0^a^
	EFV+(TDF + FTC) or (ABC+3TC) or (AZT+3TC)	338	36.4	72.2%	263^a^	5.0^a^
WAVES (Squires et al., 2016)	ATV/r + TDF + FTC	286	36	0%	385	4.56^a^
	EVG/c + TDF + FTC	289	36	0%	376	4.46^a^

ABC, abacavir; ATV, atazanavir; AZT, azidothymidine; BIC, bictegravir; c, cobicistat; DOR, doravirine; DRV, darunavir; DTG, dolutegravir; EFV, efavirenz; EVG, elvitegravir; FTC, emtricitabine; LPV, lopinavir; NVP, nevirapine; r, ritonavir; RAL, raltegravir; ROB, risk of bias; RPV, rilpivirine; TAF, tenofovir alafenamide; TDF, tenofovir disoproxil fumarate; VL, viral load; 3TC, lamivudine.

a Median.

b not analysis.

### Virologic Suppression

**FIGURE 3:**
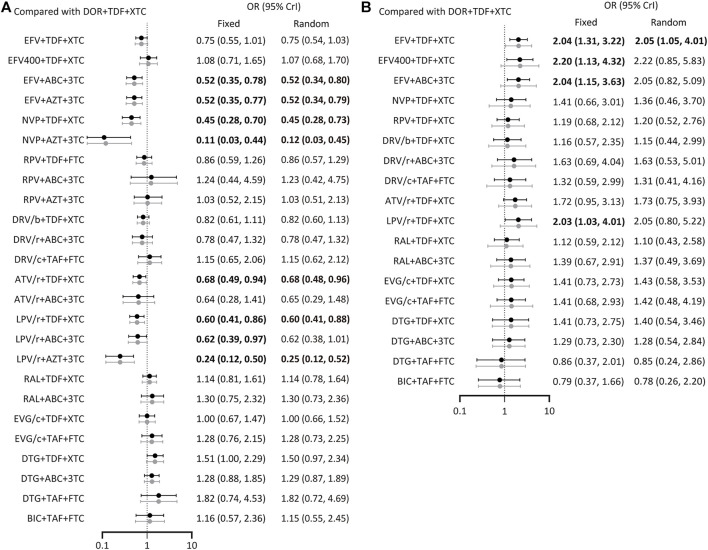
Forest plot of outcomes in terms of **(A)** virological suppression, **(B)** adverse events. Results of fixed effect model were drawn in black, and results of random effect model were drawn in grey. ABC: abacavir; ATV: atazanavir; AZT: Azidothymidine; BIC: bictegravir; c: cobicistat; DOR: doravirine; DRV: darunavir; DTG: dolutegravir; EFV: efavirenz; EVG: elvitegravir; FTC: emtricitabine; LPV: lopinavir; NVP: nevirapine; r: ritonavir; RAL: raltegravir; RPV: rilpivirine; TAF: tenofovir alafenamide; TDF: tenofovir disoproxil fumarate; XTC: emtricitabine or lamivudine; 3TC: lamivudine.

All included studies reported the number of participants with a HIV RNA load of <50 copies/mL at 48 weeks. A total of 26 treatment regimens were analyzed for this outcome. Network analysis revealed that EFV + TDF+3TC/FTC had the most connection times (Figure 2A). Fixed-effect model analysis found that DOR + TDF+3TC/FTC has a higher proportion of people achieved virological suppression relative to most NNRTI and PI-based regimens (Figure 3A), indicating good efficacy. DTG + TAF+3TC/FTC had the highest SUCRA value (SUCRA = 90.28%), indicating that it is the best regimen with regards to virological suppression, while DOR + TDF+3TC/FTC (SUCRA = 61.51%) ranked 12th, indicating relatively lower effectiveness (Table 2).

**TABLE 2:** SUCRA value.

Ranking	Virological Suppression		Adverse Events		Serious Adverse Events		Drug-related Adverse Events		Subgroup Analysis	
1	Y	90.28%	Z	88.01%	Y	92.09%	Z	98.70%	Y	87.86%
2	W	89.81%	Y	80.53%	M	86.90%	S	85.26%	T	87.55%
3	X	80.68%	A	74.55%	A	83.72%	A	84.26%	W	87.5%
4	T	79.76%	S	70.95%	K	80.75%	W	82.34%	X	79.38%
5	V	79.51%	K	66.79%	H	78.86%	Y	75.18%	M	76.73%
6	S	72.23%	H	62.88%	Z	68.16%	T	65.57%	C	74.85%
7	M	71.39%	X	58.40%	L	64.78%	X	62.34%	S	62.08%
8	I	71.29%	M	54.51%	B	58.37%	M	60.44%	Z	58.14%
9	Z	70.1%	F	52.40%	C	55.70%	F	56.03%	B	55.01%
10	C	67.35%	T	50.99%	S	51.29%	V	53.98%	A	54.68%
11	J	63.39%	W	49.34%	X	49.89%	H	50.02%	U	51.15%
12	A	61.51%	V	48.61%	W	43.10%	U	44.83%	F	45.95%
13	U	61.04%	U	48.44%	P	40.24%	K	37.09%	K	39.27%
14	H	50.13%	L	38.07%	E	36.04%	L	31.63%	V	39.26%
15	K	45.81%	N	30.58%	T	33.35%	C	23.05%	H	37.39%
16	L	42.85%	E	21.15%	U	30.74%	N	18.84%	N	26.35%
17	B	38.45%	P	18.83%	N	25.21%	P	8.42%	D	23.07%
18	N	31.82%	C	17.74%	R	22.18%	B	7.85%	L	22.49%
19	O	31.78%	B	17.22%	V	21.55%	D	4.15%	P	15.22%
20	Q	26.95%	-	-	F	16.58%	-	-	Q	13.67%
21	P	23.49%	-	-	D	10.52%	-	-	E	12.39%
22	D	17.1%	-	-	-	-	-	-	-	-
23	E	16.82%	-	-	-	-	-	-	-	-
24	F	11.56%	-	-	-	-	-	-	-	-
25	R	4.16%	-	-	-	-	-	-	-	-
26	G	0.75%	-	-	-	-	-	-	-	-

A: DOR + TDF + XTC; B: EFV + TDF + XTC; C: EFV400 + TDF + XTC; D: EFV + ABC+3TC; E: EFV + AZT+3TC; F: NVP + TDF + XTC; G: NVP + AZT+3TC; H: RPV + TDF + FTC; I: RPV + ABC+3TC; J: RPV + AZT+3TC; K: DRV/b + TDF + XTC; L: DRV/r + ABC+3TC; M: DRV/c + TAF + FTC; N: ATV/r + TDF + XTC; O: ATV/r + ABC+3TC; P: LPV/r + TDF + XTC; Q: LPV/r + ABC+3TC; R: LPV/r + AZT+3TC; S: RAL + TDF + XTC; T: RAL + ABC+3TC; U: EVG/c + TDF + XTC; V: EVG/c + TAF + FTC; W: DTG + TDF + XTC; X: DTG + ABC+3TC; Y: DTG + TAF + FTC; Z: BIC + TAF + FTC. ABC: abacavir; ATV: atazanavir; AZT: azidothymidine; BIC: bictegravir; c: cobicistat; DOR: doravirine; DRV: darunavir; DTG: dolutegravir; EFV: efavirenz; EVG: elvitegravir; FTC: emtricitabine; LPV: lopinavir; NVP: nevirapine; r: ritonavir; RAL: raltegravir; RPV: rilpivirine; TAF: tenofovir alafenamide; TDF: tenofovir disoproxil fumarate; XTC: emtricitabine or lamivudine; 3TC: lamivudine.

### Adverse Events

A total of 19 regimens reported data on adverse events (Figure 2B). Since the comparison between DTG + ABC+3TC and RAL + TDF+3TC/FTC had a *p* < 0.05 using the node splitting method, the random-effects model was applied. The incidence of adverse events with DOR + TDF+3TC/FTC was lower than with EFV + TDF+3TC/FTC and was statistically significant (OR = 2.05, 95% CrI = 1.05–4.0). Comparisons between DOR + TDF+3TC/FTC and ARTs except EFV + TDF+3TC/FTC showed no statistical differences (Figure 3B). The SUCRA value for DOR + TDF+3TC/FTC was 74.55% (third rank), indicating its incidence of adverse events maybe higher than DTG + TAF + FTC and BIC + TAF + FTC, but lower than other regimens (Table 2).

### Serious Adverse Events

**FIGURE 4:**
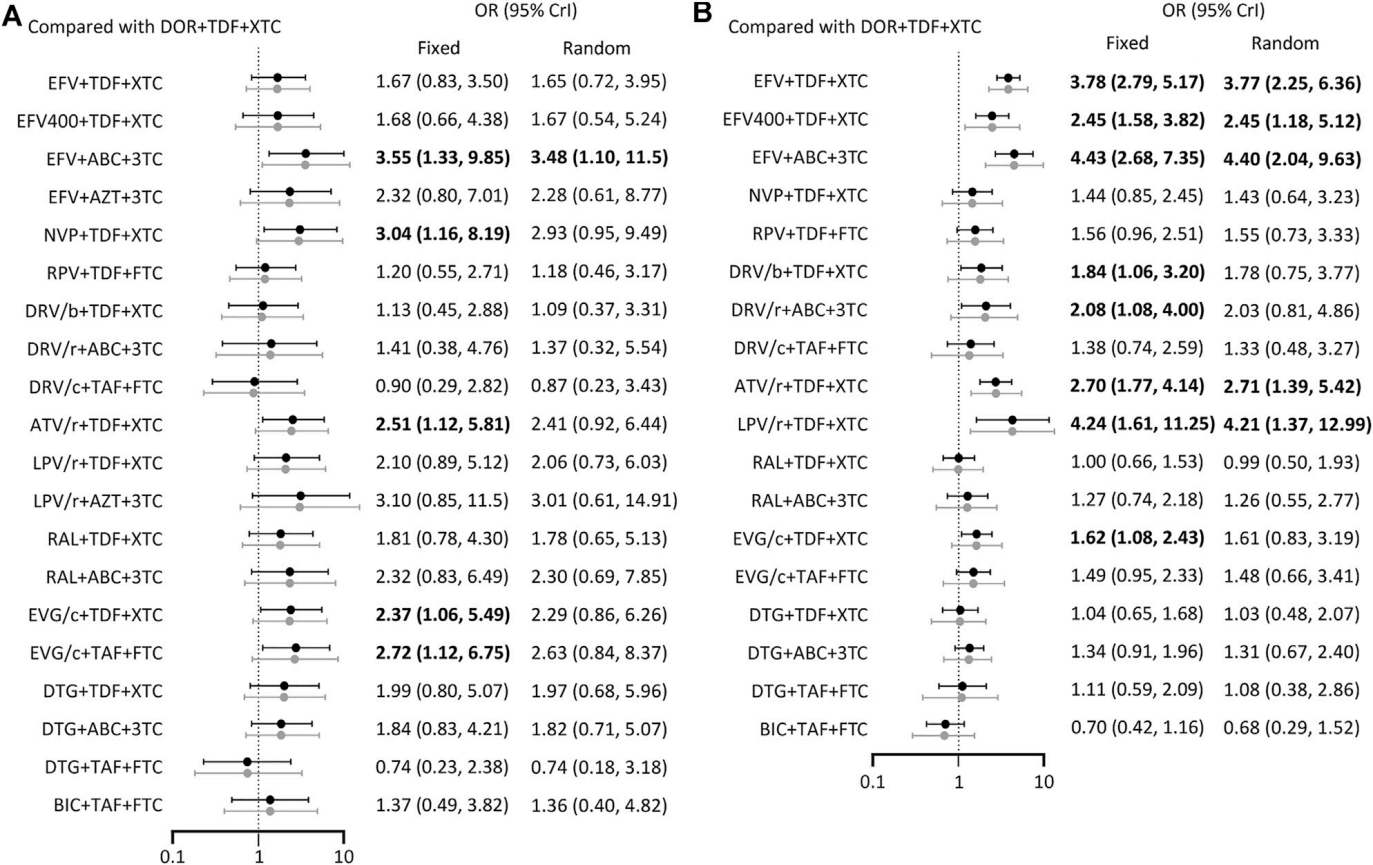
Forest plot of outcomes in terms of **(A)** serious adverse events **(B)** drug-related adverse events. Results of fixed effect model were drawn in black, and results of random effect model were drawn in grey. ABC: abacavir; ATV: atazanavir; AZT: Azidothymidine; BIC: bictegravir; c: cobicistat; DOR: doravirine; DRV: darunavir; DTG: dolutegravir; EFV: efavirenz; EVG: elvitegravir; FTC: emtricitabine; LPV: lopinavir; NVP: nevirapine; r: ritonavir; RAL: raltegravir; RPV: rilpivirine; TAF: tenofovir alafenamide; TDF: tenofovir disoproxil fumarate; XTC: emtricitabine or lamivudine; 3TC: lamivudine.

A total of 27 studies reported the occurrence of serious adverse events involving 21 treatment regimens (Figure 2C). Forest plot showed DOR + TDF+3TC/FTC was statistically different from EFV + ABC+3TC (OR = 3.55, 95% CrI = 1.33–9.85), NVP + TDF+3TC/FTC (OR = 3.04, 95% CrI = 1.16–8.19), ATV/r + TDF+3TC/FTC (OR = 2.51, 95% CrI = 1.12–5.81), EVG/c + TDF+3TC/FTC (OR = 2.37, 95% CrI = 1.06–5.49), and EVG/c + TAF + FTC (OR = 2.72, 95% CrI = 1.12–6.75). These ORs and 95% CrIs were all >1, indicating that DOR + TDF+3TC/FTC had higher safety compared with the above regimens with regards to serious adverse events (Figure 4A). Based on SUCRA value, DOR + TDF+3TC/FTC ranks third (SUCRA = 83.72%), indicating its incidence of serious adverse events maybe higher than DTG + TAF + FTC and DRV/c + TAF + FTC, but lower than other regimens (Table 2).

### Drug-Related Adverse Events

The 19 ARTs compared in the 19 trials constituted a network of evidence for drug-related adverse events (Figure 2D). DOR + TDF+3TC/FTC exhibited better safety than EFV-based regimens. Comparison between DOR + TDF+3TC/FTC and DRV-based regimens revealed different results for different backbones. ABC+3TC and TDF+3TC/FTC with DRV had more drug-related adverse events, while TAF + FTC with DRV had no statistically significant difference relative to DOR + TDF+3TC/FTC. Most of the comparisons between DOR + TDF+3TC/FTC and ARTs containing INI showed no statistical differences. Only comparison with EVG/c + TDF+3TC/FTC showed relatively poor safety of the latter (Figure 4B). The SUCRA value of DOR + TDF+3TC/FTC was 84.26%, indicating its incidence of drug-related adverse events maybe higher than BIC + TAF + FTC and RAL + TDF+3TC/FTC, but lower than other regimens (Table 2).

### Subgroup Analysis

**FIGURE 5:**
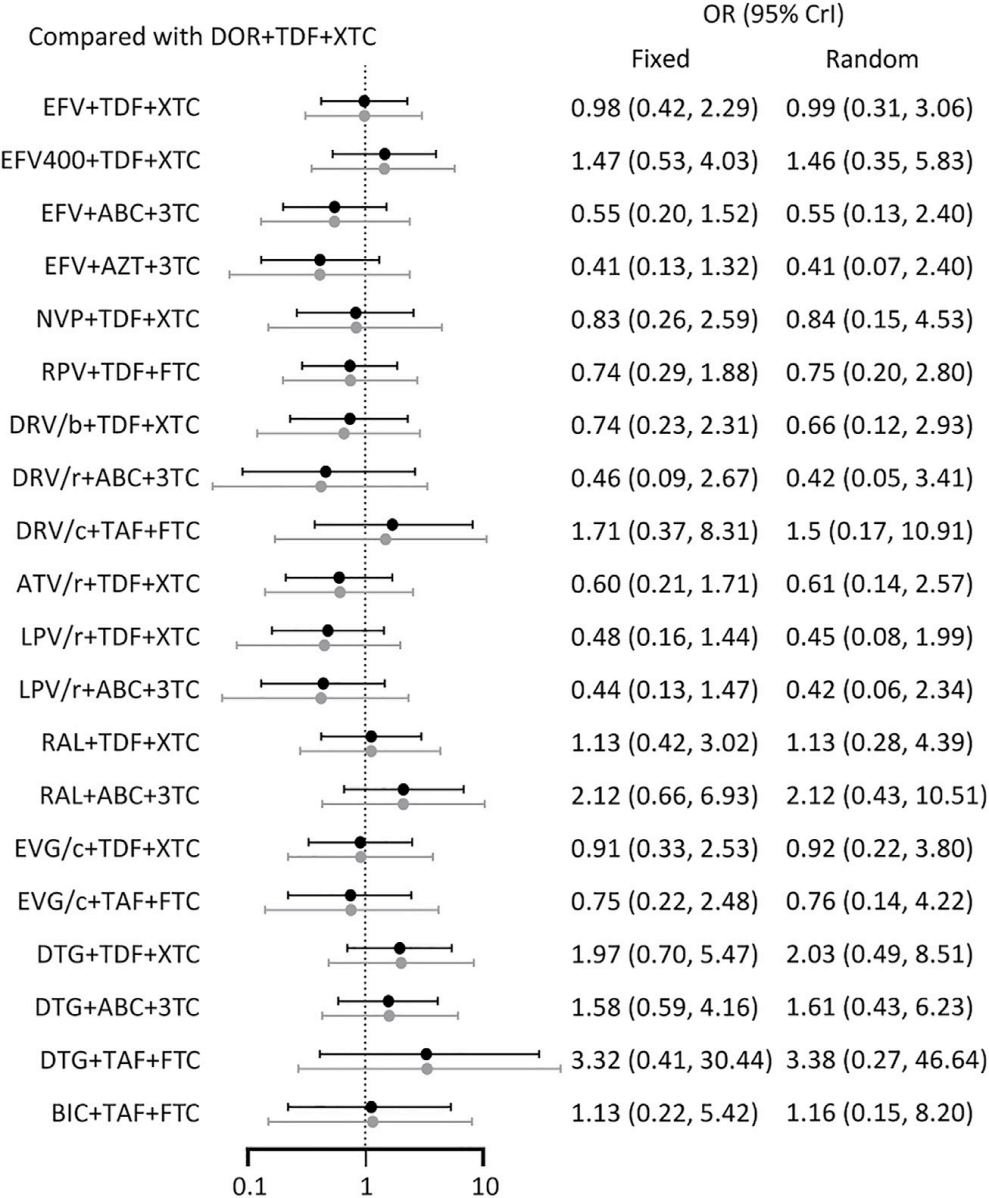
Forest plot of outcomes in terms of subgroup analysis. Results of fixed effect model were drawn in black, and results of random effect model were drawn in grey. ABC: abacavir; ATV: atazanavir; AZT: Azidothymidine; BIC: bictegravir; c: cobicistat; DOR: doravirine; DRV: darunavir; DTG: dolutegravir; EFV: efavirenz; EVG: elvitegravir; FTC: emtricitabine; LPV: lopinavir; NVP: nevirapine; r: ritonavir; RAL: raltegravir; RPV: rilpivirine; TAF: tenofovir alafenamide; TDF: tenofovir disoproxil fumarate; XTC: emtricitabine or lamivudine; 3TC: lamivudine.

With regards to virological suppression in patients with viral loads >100,000 copies/mL, all other ARTs had no statistically significant difference relative to DOR + TDF+3TC/FTC (Figure 5). SUCRA value showed that DOR + TDF+3TC/FTC ranked relatively low (Table 2). This is probably because many trials did not report results for subgroup analysis, resulting in a corresponding reduction in the number of treatment regimens (Figure 2E) and participants, thus increasing the uncertainty and CrI.

### Evidence Grading

Based on evidence grading by CINeMA, most results were rated as “moderate” and “low” (Table 3). There are two main reasons for downgrading. First, many trials were not blinded, resulting in a trial rating of “low quality,” which may affect the results of the comparison between relevant regimens, leading to the downgrade. Additionally, where CrI included 1, results were not statistically significant, and were downgraded.

**TABLE 3:** CINeMA evidence grading.

Compared with DOR + TDF + XTC	Evidence Grading				
	Virological Suppression	Adverse Events	Serious Adverse Events	Drug-related Adverse Events	Subgroup Analysis
EFV + TDF + XTC	Low	Moderate	Very low	Moderate	Very low
EFV400 + TDF + XTC	Low	High	Low	High	Low
EFV + ABC+3TC	Low	Moderate	Moderate	Moderate	Very low
EFV + AZT+3TC	Low	-	Very low	-	Very low
NVP + TDF + XTC	Low	Very low	Very low	Very low	Very low
NVP + AZT+3TC	Low	-	-	-	-
RPV + TDF + FTC	Low	Very low	Very low	Moderate	Very low
RPV + ABC+3TC	Low	-	-	-	-
RPV + AZT+3TC	Low	-	-	-	-
DRV/b + TDF + XTC	Moderate	Very low	Very low	Moderate	Very low
DRV/r + ABC+3TC	Low	Very low	Very low	Moderate	Very low
DRV/c + TAF + FTC	Low	Low	Low	Low	Low
ATV/r + TDF + XTC	Low	Low	Moderate	High	Low
ATV/r + ABC+3TC	Very low	-	-	-	
LPV/r + TDF + XTC	Low	Very low	Very low	Low	Very low
LPV/r + ABC+3TC	Very low	-	-	-	Very low
LPV/r + AZT+3TC	Low	-	Very low	-	-
RAL + TDF + XTC	Low	Low	Low	Low	Low
RAL + ABC+3TC	Low	Low	Low	Low	Low
EVG/c + TDF + XTC	Low	Low	Moderate	Moderate	Low
EVG/c + TAF + FTC	Low	Low	Moderate	Moderate	Low
DTG + TDF + XTC	Moderate	Low	Low	Low	Low
DTG + ABC+3TC	Moderate	Low	Low	Moderate	Low
DTG + TAF + FTC	Low	Low	Low	Low	Low
BIC + TAF + FTC	Low	Low	Low	Low	Low

ABC: abacavir; ATV: atazanavir; AZT: azidothymidine; b: ritonavir or cobicistat; BIC: bictegravir; c: cobicistat; DOR: doravirine; DRV: darunavir; DTG: dolutegravir; EFV: efavirenz; EVG: elvitegravir; FTC: emtricitabine; LPV: lopinavir; NVP: nevirapine; r: ritonavir; RAL: raltegravir; RPV: rilpivirine; TAF: tenofovir alafenamide; TDF: tenofovir disoproxil fumarate; XTC: emtricitabine or lamivudine; 3TC: lamivudine.

## Discussion

This study compared the efficacy and safety of DOR + TDF+3TC/FTC with traditional three-drug regiments at 48 weeks in treatment-naïve HIV-1-infected adults. Compared with cohort studies and case-control studies, RCTs are of higher grade, so this study chose to include RCTs for NMA. A total of 39 RCTs involving more than 20,000 patients were included according to the inclusion criteria, and a maximum of 26 treatments were compared simultaneously (Orkin et al., 2019; Molina et al., 2018; Lennox et al., 2014; Miro et al., 2015; Puls et al., 2010; Eron et al., 2018; Orrell et al., 2017; Ortiz et al., 2008; Soriano et al., 2011; Post et al., 2010; Molina et al., 2008; DeJesus et al., 2004; Harris et al., 2009; Landman et al., 2014; Molina et al., 2011; ENCORE1 Study Group, 2014; Nishijima et al., 2013; Clotet et al., 2014; Sax et al., 2012; DeJesus et al., 2012; Sax et al., 2015; Gallant et al., 2017; Sax et al., 2017; Smith et al., 2009; Honda et al., 2011; Echeverría et al., 2010; Aberg et al., 2012; Kouanfack et al., 2019; Dejesus et al., 2011; Mussini et al., 2019; Sierra-Madero et al., 2010; Walmsley et al., 2013; Raffi et al., 2013; Cohen et al., 2014; Lennox et al., 2009; Gallant et al., 2006; Cohen et al., 2012; Cohen et al., 2011; Squires et al., 2016). Baseline characteristics of the participants were similar. The results of this study are consistent with those of DRIVE-AHEAD and DRIVE-FORWORD (Molina et al., 2018; Orkin et al., 2019). Two phase three RCTs (DRIVE-AHEAD, DRIVE-FORWORD) have proven that DOR has good efficacy and safety in treatment-naïve HIV-1 adults (Molina et al., 2018; Orkin et al., 2019). DRIVE-FORWORD showed that compared to DRV/r (TDF + FTC or ABC+3TC backbone), DOR was not less effective in virological suppression and had similar safety (Molina et al., 2018). DRIVE-AHEAD compared DOR + TDF+3TC with EFV + TDF + FTC and uncovered similar virologic efficacy, but the safety of the former was better (Orkin et al., 2019).

In this study, in terms of virological suppression, DOR++TDF+3TC/FTC was not inferior to EFV + TDF + FTC/3TC and DRV/r-based regimens. But ORs between DOR++TDF+3TC/FTC and INI-based regimens were ≥1, indicating INI-based regimens had a higher proportion of patients achieving virological suppression. In subgroup analysis, DOR performed similarly in the high viral load population as in the full population, except that the comparison with EVG had an OR more than 1. For safety analysis, adverse events, serious adverse events, and drug-related adverse events which were commonly reported in studies were selected for analysis. In the above three outcome events, DOR performed well and SUCRA value ranked the third. It needs to be mentioned that local inconsistency was found in the analysis of adverse events (*p* < 0.05 for node splitting analysis between DTG + ABC+3TC and RAL + TDF+3TC/FTC), in other words, direct and indirect comparisons between DTG + ABC+3TC and RAL + TDF+3TC/FTC were inconsistent. We found that *I* ^*2*^ was lower than 50% by anohe analysis, considering the heterogeneity between the data is acceptable, and the inconsistency may be due to some differences in the baseline characteristics of the studies included in this outcome event.

With the continuous development of ART, HIV infection has changed from a deadly disease to a chronic long-term disease. Although AIDS cannot be cured at present, ART can significantly reduce the viral loads, maintain patients’ immune function, and prolong life expectancy (Antiretroviral Therapy Cohort Collaboration, 2017; Wu, 2016; Marcus et al., 2000; GBD 2019 HIV Collaborators, 2021; Smiley et al., 2021). In recent years, the role of NNRTIs in ART has been weakened to some extent with the emergence of more and more tolerated antiretroviral drugs and regimens. DOR is a new NNRTI, which can be used as a single pill with other anti-retroviral drugs, or as a fixed-dose combination with 3TC and TDF. This study showed that DOR + TDF+3TC/FTC has good efficacy and safety at 48 weeks. Additionally, the marketing of DOR + TDF+3TC as a fixed-dose combination may improve patient compliance as fewer drugs need to be taken using this regimen (Godman et al., 2020). These findings support the use of DOR + TDF+3TC/FTC in treatment-naive HIV-positive adults.

This study has some limitations. Some of the regimens included in NMA to analyze certain outcome events lacked the data required for analysis and were not included in the study. Additionally, immune-reconstitution (recovery of CD4^+^ T-cells) is also an important indicator for evaluating the efficacy of treatment regimens in HIV-infected patients after initiating ART. However, due to inconsistent reported data units, the comprehensive analysis could not be conducted. Thus, we did not compare DOR + TDF+3TC/FTC with other ARTs in immune-reconstitution. Finally, CINeMA evidence grading uses R and the netmeta package, which adopts the frequency method for statistical analysis and may differ from the Bayesian approach. Despite its limitations, NMA provided valuable evidence to support the use of DOR + TDF+3TC/FTC in untreated HIV-1-infected adults.

## Conclusion

HIV/AIDS, a human immune system disorder caused by HIV infection, is mainly treated with ART. DOR is a newly-approved antiretroviral drug belonging to the class of non-nucleoside reverse transcriptase inhibitors. Two phase 3 RCTs have proven that DOR has good efficacy and safety in HIV-1 patients. In this Bayesian network meta-analysis, we compared the efficacy and safety of DOR + TDF+3TC/FTC with traditional triple therapies in treatment-naïve HIV-1-positive patients and found that DOR + TDF+3TC/FTC has good efficacy and safety at 48 weeks.

## Data Availability

The original contributions presented in the study are included in the article/Supplementary Material, further inquiries can be directed to the corresponding authors.
