# Supplementary material for: Comparison of the Efficacy and Safety of a Doravirine-Based, Three-Drug Regimen in Treatment-Naïve HIV-1 Positive Adults: A Bayesian Network Meta-Analysis
Source: Front Pharmacol. 2022 Apr 20;13:676831. doi: 10.3389/fphar.2022.676831 (PMC9065253; doi:10.3389/fphar.2022.676831)
Supplement: Supplementary file 1 [file DataSheet1.docx]

Supplementary Material

# Supplementary Tables

**Supplementary Table S1.** Data extracted from the included studies

| **Trial** | **Treatment** | **Virological suppression, n/N** | **Subgroup analysis, n/N** | **Adverse events, n/N** | **Serious adverse events, n/N** | **drug-related adverse events, n/N** |
| --- | --- | --- | --- | --- | --- | --- |
| ACTG A5257 | ATV/r+TDF+FTC | 442/605 | - | - | - | - |
|  | RAL+TDF+FTC | 500/603 | - | - | - | - |
|  | DRV/r+TDF+FTC | 463/601 | - | - | - | - |
| Advanz-3 | EFV+TDF+FTC | 18/28 | - | 25/28 | 2/28 | 13/28 |
|  | ATV/r+TDF+FTC | 17/30 | - | 22/30 | 6/30 | 11/30 |
|  | LPV/r+TDF+FTC | 15/29 | - | 24/29 | 6/29 | 14/29 |
| Altair | EFV+FTC+TDF | 97/114 | - | 99/114 | 14/114 | - |
|  | ATV/r+TDF+FTC | 93/105 | - | 95/105 | 8/105 | - |
| AMBER | DRV/c+TAF+FTC | 331/362 | 53/59 | 312/362 | 17/362 | 126/362 |
|  | DRV/c+TDF+FTC | 321/363 | 56/70 | 307/363 | 21/363 | 151/363 |
| ARIA | DTG+ABC+3TC | 203/248 | 55/69 | 195/248 | 12/248 | 83/248 |
|  | ATV/r+TDF+FTC | 176/247 | 42/66 | 197/247 | 20/247 | 121/247 |
| ARTEMIS | DRV/r+TDF+FTC | 288/343 | 93/117 | 309/343 | 25/343 | - |
|  | LPV/r+TDF+FTC | 271/346 | 80/120 | 328/346 | 41/346 | - |
| ARTEN | ATV/r+TDF+FTC | 152/193 | 60/115 | 167/193 | 17/193 | 94/193 |
|  | NVP+TDF+FTC | 253/376 | 138/230 | 323/376 | 36/376 | 130/376 |
| ASSERT | EFV+ABC+3TC | 114/192 | 53/97 | - | 27/192 | 98/192 |
|  | EFV+TDF+FTC | 137/193 | 75/110 | - | 14/193 | 91/193 |
| CASTLE | ATV/r+TDF+FTC | 343/440 | 165/223 | 400/440 | 57/441 | - |
|  | LPV/r+TDF+FTC | 338/443 | 162/225 | 399/437 | 48/437 | - |
| CNA30024 | EFV+AZT+3TC | 224/325 | 90/126 | - | 46/325 | - |
|  | EFV+ABC+3TC | 226/324 | 97/126 | - | 65/324 | - |
| CTN177 | LPV/r+AZT+3TC | 17/25 | - | - | - | - |
|  | NVP+AZT+3TC | 13/26 | - | - | - | - |
| DAYANA | NVP+TDF+FTC | 24/31 | - | - | - | - |
|  | EFV+TDF+FTC | 21/30 | - | - | - | - |
| DRIVE-AHEAD | DOR+TDF+3TC | 307/364 | 56/69 | 301/364 | 13/364 | 113/364 |
|  | EFV+TDF+FTC | 294/364 | 59/73 | 330/364 | 21/364 | 229/364 |
| DRIVE-FORWARD | DOR+TDF+FTC | 278/333 | - | - | - | - |
|  | DRV/r+TDF+FTC | 270/335 | - | - | - | - |
|  | DRV/r+ABC+3TC | 36/48 | - | - | - | - |
| ECHO | RPV+TDF+FTC | 285/346 | 125/165 | 303/346 | 23/346 | 55/346 |
|  | EFV+TDF+FTC | 281/344 | 149/181 | 317/344 | 31/344 | 108/344 |
| ENCORE1 | EFV400+TDF+FTC | 266/321 | 86/107 | 286/321 | 23/321 | 118/321 |
|  | EFV+TDF+FTC | 242/309 | 83/107 | 273/309 | 22/309 | 146/309 |
| Epzicom-Truvada | ATV/r+TDF+FTC | 44/55 | - | - | - | - |
|  | ATV/r+ABC+3TC | 44/54 | - | - | - | - |
| FLAMINGO | DTG+ABC+3TC | 71/79 | 12/13 | 67/80 | 10/79 | 27/79 |
|  | DTG+TDF+FTC | 146/163 | 45/48 | 138/162 | 16/163 | 53/163 |
|  | DRV/r+ABC+3TC | 68/80 | 8/12 | 69/79 | 6/80 | 39/80 |
|  | DRV/r+TDF+FTC | 132/162 | 35/49 | 137/163 | 7/162 | 77/162 |
| GS-US-236-0102 | EVG/c+TDF+FTC | 305/348 | 99/118 | 327/348 | 41/348 | 161/348 |
|  | EFV+TDF+FTC | 296/352 | 95/116 | 334/352 | 24/352 | 237/352 |
| GS-US-236-0103 | EVG/c+TDF+FTC | 316/353 | 128/150 | 323/353 | 26/353 | 159/353 |
|  | ATV/r+TDF+FTC | 308/355 | 116/141 | 333/355 | 31/355 | 203/355 |
| GS-US-292-0104/0111 | EVG/c+TAF+FTC | 800/866 | 171/196 | 779/866 | 69/866 | 346/866 |
|  | EVG/c+TDF+FTC | 784/866 | 174/195 | 780/867 | 61/866 | 364/866 |
| GS-US-380-1489 | BIC+TAF+FTC | 290/314 | 46/53 | 265/314 | 19/314 | 82/314 |
|  | DTG+ABC+3TC | 293/315 | 45/50 | 283/315 | 25/315 | 127/315 |
| GS-US-380–1490 | BIC+TAF+FTC | 286/320 | 57/66 | 264/320 | 39/320 | 57/320 |
|  | DTG+TAF+FTC | 302/325 | 51/54 | 272/325 | 23/325 | 83/325 |
| HEAT | LPV/r+ABC+3TC | 232/343 | 98/155 | - | - | - |
|  | LPV/r+TDF+FTC | 232/345 | 91/140 | - | - | - |
| Japanese Anti-HIV-1 QD Therapy | EFV+ABC+3TC | 28/36 | - | - | - | - |
|  | ATV/r+ABC+3TC | 27/35 | - | - | - | - |
| Lake Study | EFV+ABC+3TC | 36/63 | - | - | - | - |
|  | LPV/r+ABC+3TC | 40/63 | - | - | - | - |
| METABOLIK | DRV/r+TDF+FTC | 26/34 | - | 31/34 | 5/34 | 15/34 |
|  | ATV/r+TDF+FTC | 22/31 | - | 29/31 | 5/31 | 22/31 |
| NAMSAL | DTG+TDF+3TC | 231/310 | 137/207 | - | - | - |
|  | EFV400+TDF+3TC | 209/303 | 123/200 | - | - | - |
| NEWART | ATV/r+TDF+FTC | 48/77 | - | 73/77 | 7/77 | 42/77 |
|  | NVP+TDF+FTC | 42/75 | - | 66/75 | 10/75 | 27/75 |
| PRADAR study | DRV/r+ABC+3TC | 16/24 | - | 19/24 | - | - |
|  | RAL+ABC+3TC | 17/22 | - | 17/22 | - | - |
| Sierra-Madero et al., 2010 | EFV+AZT+3TC | 67/95 | - | - | 17/95 | - |
|  | LPV/r+AZT+3TC | 50/94 | - | - | 21/94 | - |
| SINGLE | DTG+ABC+3TC | 364/414 | 111/134 | 369/414 | 37/414 | 180/414 |
|  | EFV+TDF+FTC | 338/419 | 100/131 | 387/419 | 35/419 | 278/419 |
| SPRING-2 | DTG+ABC+3TC | 145/169 | 30/37 | 135/169 | 11/169 | 51/169 |
|  | DTG+TDF+FTC | 216/242 | 32/39 | 204/242 | 18/242 | 65/242 |
|  | RAL+ABC+3TC | 142/164 | 64/77 | 137/164 | 14/164 | 51/164 |
|  | RAL+TDF+FTC | 209/247 | 55/77 | 203/247 | 17/247 | 68/247 |
| STaR | RPV+TDF+FTC | 338/394 | 107/134 | 349/394 | 36/394 | - |
|  | EFV+TDF+FTC | 320/392 | 116/142 | 365/392 | 48/392 | - |
| STARTMRK | RAL+TDF+FTC | 241/281 | 130/154 | 253/281 | 28/281 | 124/281 |
|  | EFV+TDF+FTC | 230/282 | 116/143 | 272/282 | 27/282 | 217/282 |
| Study 934 | EFV+TDF+FTC | 196/255 | - | 163/257 | - | - |
|  | EFV+AZT+3TC | 173/254 | - | 161/254 | - | - |
| THRIVE | RPV+TDF+FTC | 172/204 | - | - | - | - |
|  | RPV+ABC+3TC | 31/35 | - | - | - | - |
|  | EFV+TDF+FTC | 165/202 | - | - | - | - |
|  | EFV+ABC+3TC | 28/33 | - | - | - | - |
|  | RPV+AZT+3TC | 88/101 | - | - | - | - |
|  | EFV+AZT+3TC | 83/103 | - | - | - | - |
| WAVES | ATV/r+TDF+FTC | 252/289 | - | - | 24/289 | - |
|  | EVG/c+TDF+FTC | 231/286 | - | - | 29/286 | - |

**Supplementary Table S2.** Cochrane risk of bias

| Trial | Sequence generation | Allocation concealment | Blinding | Incomplete outcome data | Selective outcome reporting | Other sources of bias |
| --- | --- | --- | --- | --- | --- | --- |
| ACTG A5257 | Low | Low | High | Low | Low | Low |
| Advanz-3 | Low | Low | High | High | Low | Unclear |
| ALTAIR | Low | Low | High | Low | Low | Low |
| AMBER | Low | Low | Low | Low | Low | Low |
| ARIA | Low | Low | High | Low | Low | Unclear |
| ARTEMIS | Low | Low | High | Low | Low | Low |
| ARTEN | Unclear | Low | High | Low | Low | Low |
| ASSERT | Unclear | Low | High | High | Low | Low |
| CASTLE | Low | Low | High | Low | Low | Low |
| CNA30024 | Unclear | Low | Low | Low | Low | Low |
| CTN177 | Low | Low | High | Low | Unclear | Low |
| DAYANA | Low | Low | High | Low | Unclear | Low |
| DRIVE AHEAD | Unclear | Unclear | Low | Unclear | Unclear | Unclear |
| DRIVE FORWARD | Low | Low | Low | Low | Low | Low |
| ECHO | Low | Low | Low | Low | Low | Low |
| ENCORE1 | Low | Low | Low | Low | Low | Low |
| Epzicom-Truvada | Low | Low | High | Low | Low | Low |
| FLAMINGO | Low | Low | High | Low | Low | Low |
| GS-US-236-0102 | Low | Low | Low | Low | Low | Low |
| GS-US-236-0103 | Low | Low | Low | Low | Low | Low |
| GS-US-292-0104/0111 | Low | Low | Low | Low | Low | Low |
| GS-US-380-1489 | Low | Low | Low | Low | Low | Low |
| GS-US-380-1490 | Low | Low | Low | Low | Low | Low |
| HEAT | Unclear | Low | Low | High | Low | Low |
| Japanese Anti-HIV-1 QD Therapy | Unclear | Low | High | Low | Unclear | Low |
| LAKE Study | Unclear | Low | High | High | Low | Low |
| METABOLIK | Unclear | Low | High | Low | Low | Low |
| NAMSAL | Low | Unclear | High | Unclear | Unclear | Unclear |
| NEWART | Low | Low | High | Low | Low | Low |
| PRADAR | Unclear | Unclear | High | Low | Low | Low |
| Sierra-Madero et al., 2010 | Low | Low | High | High | Low | Low |
| SINGLE | Low | Low | Low | Low | Low | Low |
| SPRING-2 | Low | Low | Low | Low | Low | Low |
| STaR | Low | Low | High | Low | Low | Low |
| STARTMRK | Low | Low | Low | Low | Low | Low |
| Study 934 | Low | Low | High | High | Low | Low |
| THRIVE | Low | Low | Low | Low | Low | Low |
| WAVES | Low | Low | Low | Low | Low | Low |
